# Case Report: Occurrence of Severe Thoracic Aortic Aneurysms (Involving the Ascending, Arch, and Descending Segments) as a Result of Fibulin-4 Deficiency: A Rare Pathology With Successful Management

**DOI:** 10.3389/fcvm.2021.756765

**Published:** 2021-11-24

**Authors:** Paul Thomas, Aparna Venugopalan, Siddharth Narayanan, Thomas Mathew, Lakshmi Parvathi Deepti Cherukuwada, Shilpa Chandran, Jithu Pradeep, Timothy P. Fitzgibbons, Vijo George

**Affiliations:** ^1^Department of Cardiology, Government General Hospital, Ernakulam, India; ^2^Department of Pediatrics, Nationwide Children's Hospital, Columbus, OH, United States; ^3^Department of Cardiothoracic and Vascular Surgery, Government Medical College, Kottayam, India; ^4^Department of Neurology, Glenn Biggs Institute, University of Texas (UT) Health San Antonio, San Antonio, TX, United States; ^5^Department of Radiodiagnosis, Government Medical College, Thiruvananthapuram, India; ^6^Department of Internal Medicine, Montefiore Hospitals, Albert Einstein College of Medicine, Bronx, NY, United States; ^7^Department of Cardiology, University of Massachussetts Medical School, Worcester, MA, United States

**Keywords:** aneurysm, aortic disease, ascending aorta, cutis laxa, genetic predisposition, autosomal recessive, cardio-pathology

## Abstract

Aortic diseases requiring surgery in childhood are distinctive and rare. Very few reports in the literature account for the occurrence of multiple thoracic aortic aneurysms in the same pediatric patient because of a genetic cause. We report a rare occurrence of severe thoracic aortic aneurysms (involving the ascending, arch and descending aortic segments) with severe aortic insufficiency in a 7-year-old female child secondary to the extremely rare and often lethal genetic disorder, cutis laxa. She was eventually identified as a carrier of a homozygous *EFEMP2* (alias *FBLN4*) mutation. This gene encodes the extracellular matrix protein fibulin-4, and its mutation is associated with autosomal recessive cutis laxa type 1B that leads to severe aortopathy with aneurysm formation and vascular tortuosity. Parents of the child were not known to be consanguineous. Significant symptomatic improvement in the patient could be discerned after timely intervention with the valve-sparing aortic root replacement (David V procedure) and a concomitant aortic arch replacement. This is a unique report with a successful outcome that highlights the occurrence of a rare hereditary aortopathy associated with a high morbidity and mortality, and the importance of an early diagnosis and timely management. It also offers insight to physicians in having a very broad differential and multimodal approach in handling rare pediatric cardio-pathologies with a genetic predisposition.

## Introduction

Aortic diseases (aneurysm, dissection, and coarctation) and the associated valvular pathologies although rare, are significant causes of morbidity and mortality in children and young adults (1). Aortic aneurysm is a localized dilation of the aorta and may be classified as either true (dilated segment involving all the three vessel lumen layers) or false (pseudoaneurysm) (2). It can be further classified according to its morphology as fusiform or saccular and can develop both in the thoracic and abdominal aorta. Several conditions cause an aneurysm including atherosclerosis, connective tissue disorders (such as Marfan syndrome, Ehlers-Danlos syndrome), bicuspid aortic valve disease, infection, and idiopathic reasons (2).

Thoracic aortic aneurysms (TAA) are quite atypical within the pediatric population and customarily present in conjunction with antecedent diagnosis of some connective tissue disorder (3). Patients are primarily classified into two categories, those having aortic root and/or ascending aortic dilatation usually linked with aortic insufficiency (AI), and those having supra-coronary dilatation of the ascending aorta with or without AI (3, 4). Management of TAA fundamentally relies on the extent of aortic root involvement, considering the morphology of the aortic valve leaflets.

While a hereditary predisposition to TAA very strongly increases the risk of aortopathy to all segments of the vessel, pathogenic mechanisms can vary depending on the specific aortic location (5). Mutations that cause TAA interfere with the function of genes that encode components of the extracellular matrix (ECM) or proteins implicated in the transduction of either mechanical or biochemical signals in vascular smooth muscle cells (5). The major spectrum of heritable connective tissue diseases related to aortopathy tend to follow an autosomal dominant pattern of inheritance but some mutations (such as those in the *EFEMP2* gene, alias *FBLN4*) that result in aneurysms are autosomal recessive in nature (6).

*EFEMP2* encodes an ECM protein fibulin-4, which is involved in the elastic fiber assembly and function and is related to smooth muscle cell differentiation and aortic contractility (7). Mutations in *EFEMP2* are associated with the disease phenotype autosomal recessive cutis laxa type 1B (ARCL 1B, MIM number: 614437) (5). ARCL 1B is extremely rare, results in high lethality in early childhood and can be complicated by aortic aneurysm and vascular tortuosity in addition to cutis laxa (8). Paramount importance is given to the symptoms and its detrimental impact on the quality of life. The current report presents an unusual occurrence of multiple TAA (involving the ascending, arch, and the descending segments) with severe AI in a pediatric patient who was eventually identified having fibulin-4 deficiency (point mutation in *EFEMP2* gene). The patient was successfully managed and discharged after incorporating timely multimodal interventions.

## Case

A 7-year-old female child was admitted to our hospital (Government General Hospital, Ernakulam) with complaints of dyspnoea on exertion and palpitations. She was apparently normal and even active in sports, a month prior to the development of her symptoms. She was born by full-term normal vaginal delivery and had normal birth weight with no relevant past medical history.

**Figure 1:**
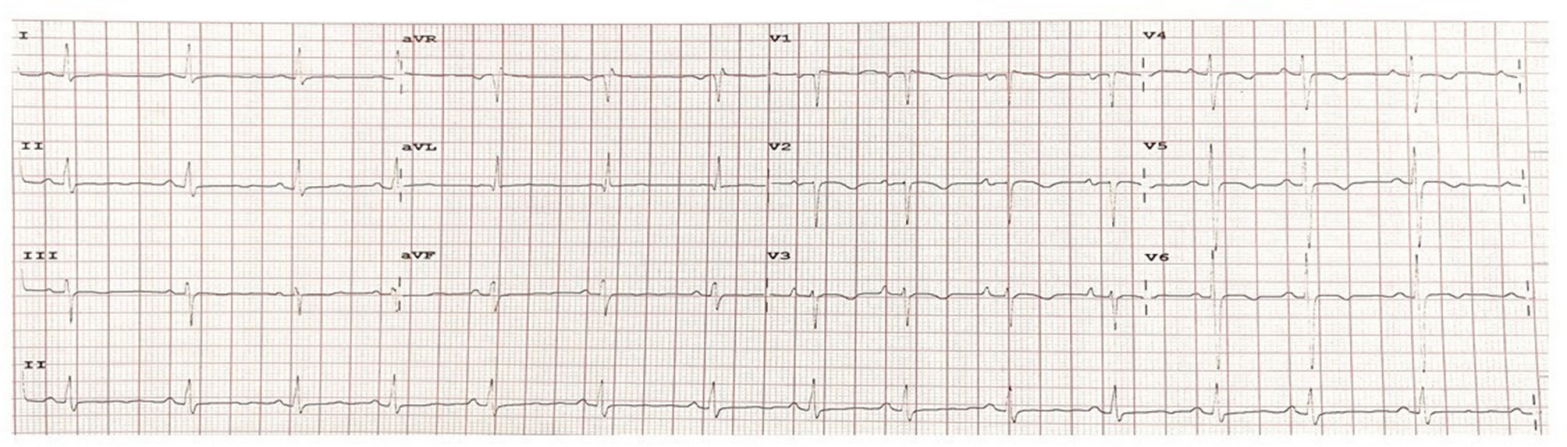
A pre-operative electrocardiogram showed sinus rhythm with a heart rate of 75 beats per minute and p mitrale (noted by the negative q wave deflection of more than 1 mm in the lead V1, suggestive of left atrial enlargement).

Initial physical examination at the pediatric outpatient department revealed no dysmorphic features apart from soft skin that was not striking, and a high-volume collapsing pulse. A blood pressure of 120/50 mmHg was recorded in bilateral arms. Significant findings from a cardiovascular examination included a precordial bulge, multiple visible pulsations over the precordium, gross cardiomegaly with hyperdynamic apex palpable over the left 5th intercostal space, early diastolic murmur over the second intercostal space/aortic area, and pansystolic murmur over the apex upon auscultation. The electrocardiogram showed sinus rhythm with a rate of 75 per minute and p mitrale (Figure 1). Upon a cardiology consultation, her echocardiography revealed situs solitus, levocardia, normal AV-VA concordance, intact inter-atrial septum and ventricular septum, good biventricular function, aneurysmal dilatation of the ascending aorta and the arch, severe AI, mild mitral regurgitation with no patent ductus arteriosus, a trileaflet AV without vegetations, no coarctation, and normal left ventricular (LV) ejection fraction.

**Figure 2:**
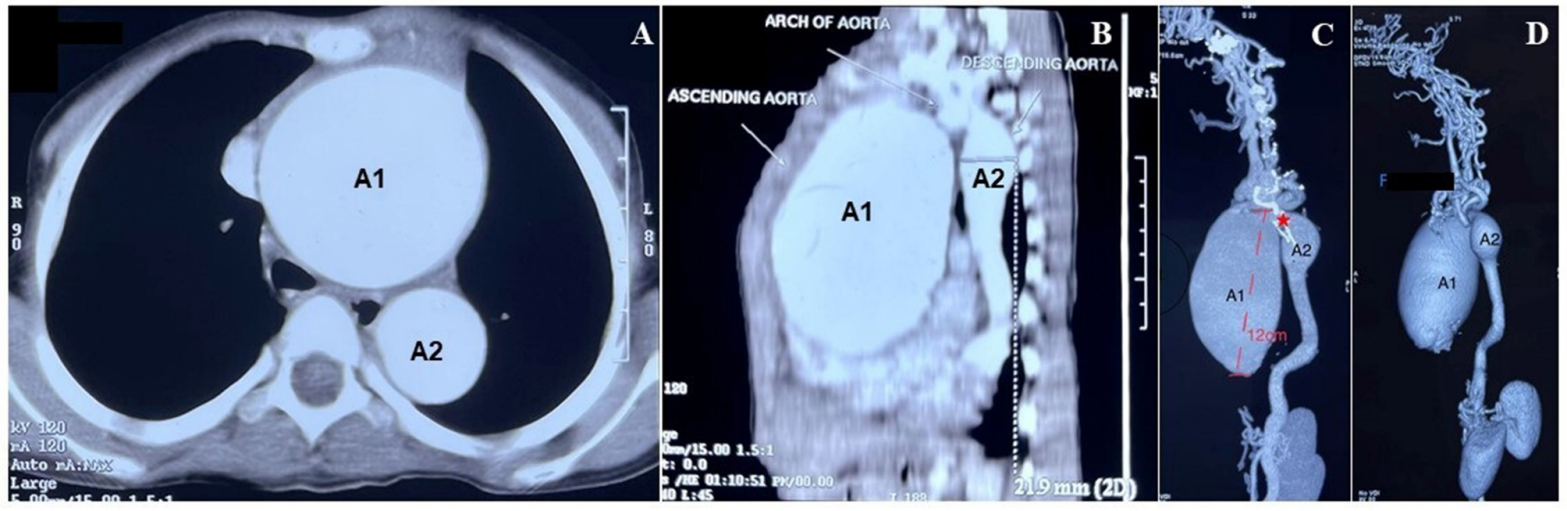
Diagnosis of two fusiform aortic aneurysms. **(A)** Computerized tomography (CT) aortogram section (axial view) below the level of the aortic arch showed marked dilatation of the ascending (A1) and the descending (A2) aorta (scale 1:5). **(B)** CT aortogram shows the sagittal view of the dilated aneurysms as described in **(A)**. **(C)** Reconstructed maximum intensity projection images of a contrast-enhanced CT showed 2 fusiform aneurysms. The larger aneurysm (A1) ranges for a length of about 12 cm, involving the ascending aorta and extending to proximal arch along with root involvement. The smaller (A2) aneurysm was noted extending from the distal arch to the proximal descending aorta (scale 1:5). The prominent “sausage link appearance” (*), can be appreciated between the 2 aneurysms. The rest of the descending aorta showed a tortuous course, atypical for the age of the patient. **(D)** CT images (3D volume rendered) show the aneurysms as described in **(B)**.

A computerized tomography (CT) aortogram revealed two fusiform aortic aneurysms involving the ascending aorta and arch, and another involving the distal arch and the proximal descending thoracic aorta with cardiomegaly (Figure 2A). A “sausage link” appearance of the aneurysmal aorta together with the observed arterial tortuosity raised a strong suspicion for hereditary aortopathy (Figures 2B–D). Parents of the child were not known to be consanguineous. A CT angiogram of the head detected no aneurysms or any other abnormal findings. The laboratory investigations (blood and urine) including troponin levels were within normal limits. Blood cultures taken prior to the initiation of any treatment were sterile after 5 days of incubation. Gene sequencing analysis showed negative findings for mutations in FBN1, FBN2, TGFBR2, TGFBR1, ACTA2, and SLC2A10 but a positive finding for a mutation in EFEMP2. A homozygous missense variation in exon 7 of the EFEMP2 gene (c. 608A>C) resulted in an amino acid substitution of alanine for aspartic acid at codon 203 (p. Asp203Ala), indicative of an autosomal recessive pathogenic variant of cutis laxa type 1B.

**Figure 3:**
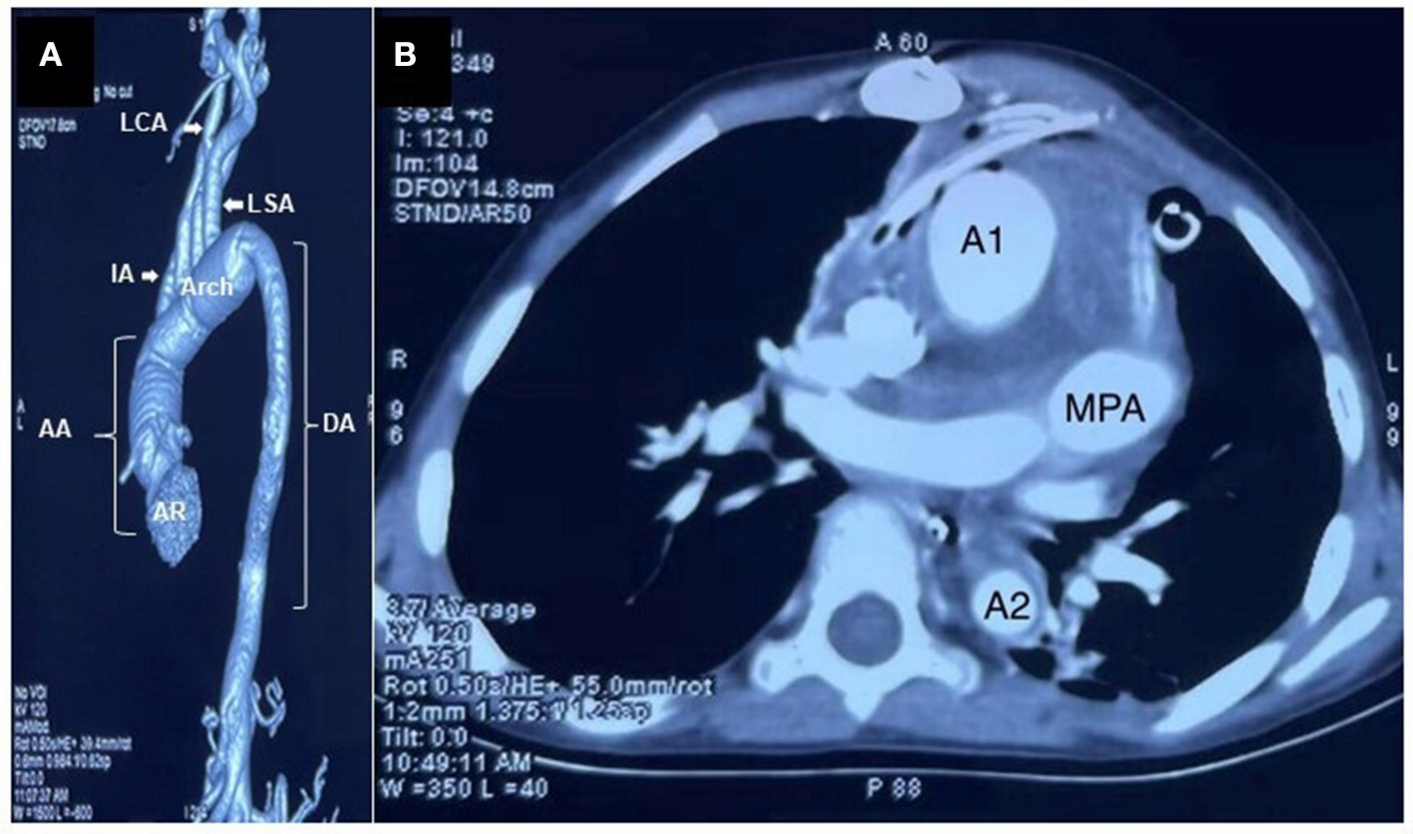
Images after the valve-sparing operation along with an aortic arch replacement. **(A)** Contrast-enhanced CT image (volume rendered) showed significant reduction in the caliber of the aorta (AR—aortic root, AA—Ascending Aorta, DA—Descending Aorta, LCA—Left common Carotid Artery, LSA—Left Subclavian Artery, IA—Innominate Artery). **(B)** Post valve-sparing surgery along with the aortic arch replacement, a CT aortogram section (axial view) at the level and just below the main pulmonary artery (MPA) showed significant reduction in the caliber of ascending aorta (A1) and descending aorta (A2). Minimally enhancing soft tissue thickening was noted surrounding the great vessels, suggestive of postoperative changes (scale 1:5).

**Figure 4:**
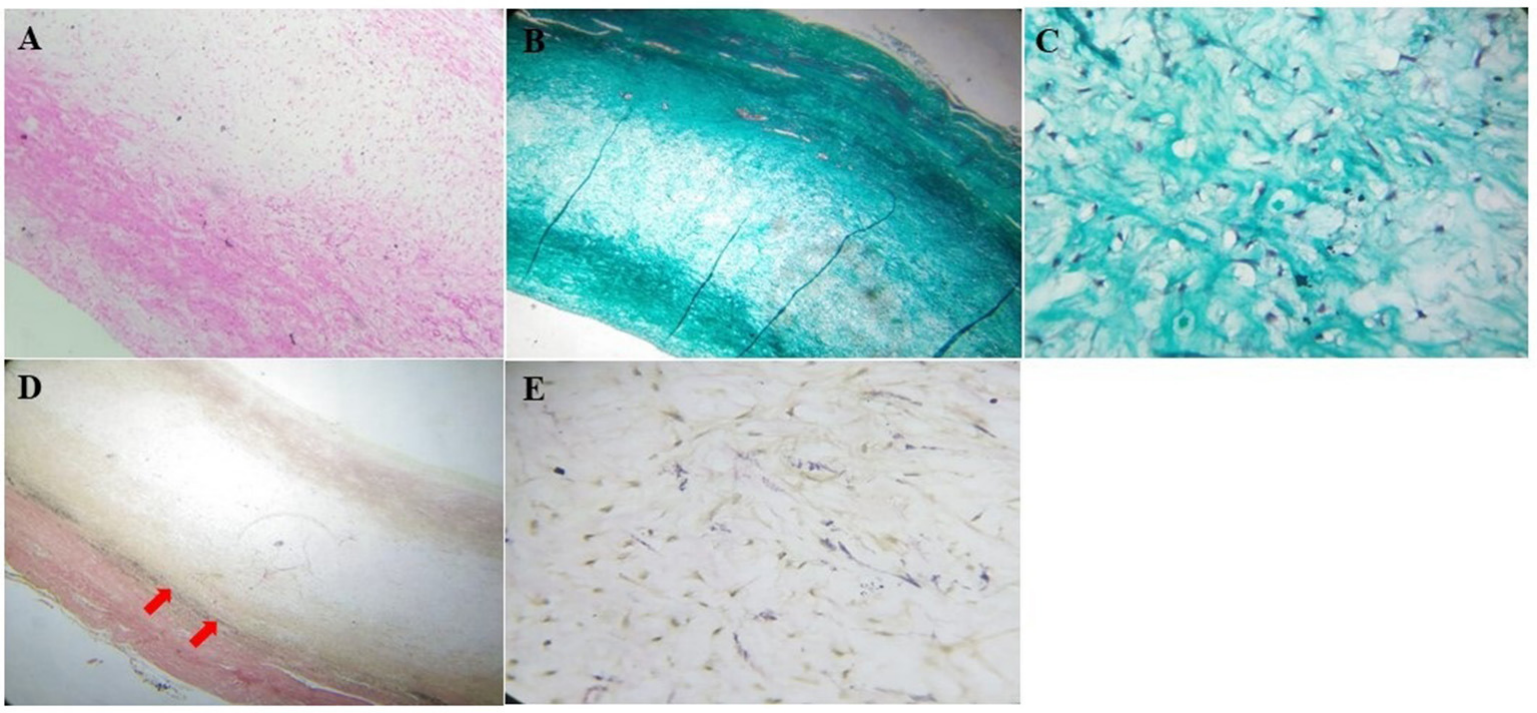
Histopathological assessment of the resected aorta. **(A)** The wall of the aorta stained with hematoxylin and eosin stain showed thickened tunica media with myxoid change (100x). **(B)** Masson's trichome staining showed disorganization and fragmentation of collagen (40x) with, **(C)** an increase in fibroblasts in the tunica media (400x). **(D)** Verhoeff-van Gieson stain showed deficient and fragmented elastic fibers (red arrows, 40x), with **(E)** increased fibroblasts (400x).

The patient was admitted for cardio-thoracic surgical intervention. She underwent a valve-sparing (David V procedure) operation along with an aortic arch replacement (Figure 3). Histopathological assessments of the resected tissue samples demonstrated disorganization and fragmentation of elastin and collagen fibers (Figure 4). Intra-operative and postoperative periods were uneventful. Anterior mediastinoscopy and drainage of mediastinal collections were performed after 2 weeks. Post-operative blood, urine and mediastinal cultures were negative. In view of low-grade fever and mediastinal collections, empirical antibiotics (intravenous meropenem and amikacin) were given for a period of 36 days post-operatively. Her post-operative echocardiogram revealed moderate AI, mild mitral regurgitation, mild to moderate LV dysfunction and aortic valve area (1.7 cm^2^, *p* = 11 mmHg), negligible tricuspid regurgitation and no pulmonary arterial hypertension. The patient improved symptomatically after the surgery but was retained in the hospital for over 2 months in view of the prevailing COVID-19 pandemic. She was prescribed aspirin (325 mg), furosemide (5 mg), and spironolactone (12.5 mg) and was eventually discharged under good medical conditions, with heeded caution to have systematic, life-long follow-ups.

## Discussion

TAA are very rare in the pediatric population and have primarily been ascribed to autosomal dominant conditions such as the Loeys-Dietz, osteoartheritis-aortopathy, and the neonatal Marfan syndromes (6). The aneurysms in these conditions typically involve uneven dilation of the aortic root (9). In contrast, our patient presented with severe dilatation of the ascending aorta, the descending aorta, and the arch, a unique observation when comparing the 42 published cases on TAA due to ARCL 1B (Table 1, reports published in English language only). Our patient was identified having an *EFEMP2* mutation that linked her aortopathy to the ARCL 1B disease. *EFEMP2* encodes for the ECM protein fibulin-4. Deficiency of fibulin-4, an elastin-binding ECM protein prominently expressed in the medial layer of large veins, arteries and skin, affects the expression and localization of fibrillary collagen, leading to elastic fiber disarray and aortic aneurysm formation (6, 8, 14).

**Table 1:** Summary of articles (12 including the current study) reporting occurrence of thoracic aortic aneurysms due to fibulin-4 deficiency (ARCL 1B)^#^.

**Paper/article type/references/year**	**Age/sex**	**Aneurysm characteristics (ECG)**	**Genetic screen**	**Resected aorta histology (yes/no)**	**Survival**
Current case report/2021	7 years, F	AA + AAr + DA	c. 608A>C, p. Asp203Ala, HZ missense mutation	No	Long-term survival (stable during follow-up, one year post surgery)
Yetman et al./Case report/(6)/2019	3 years, M	Dilation of the AA + AAr + size discrepancy between AA and DA.	c. 409A>T, p. Ser137Cys, HZ missense mutation	No (mentioned in next but data not shown)	Stable during the follow-up at two years after surgery
Sulu et al./Case series/ (10) /2019	5 patients—members of the same family	All patients identified having dilation of AR ± AA	c. 1189G>A, p. Ala397Thr HZ missense mutation	No	4/5 children survive (when report published); 3 who underwent surgery were stable
Hibino et al./Case report/(7) /2018	4-months, M	AA + DA	Compound HtZ mutation (does not mention the specific mutation type)	Yes	Surgery performed at 33 months of age. A postoperative CE-CT confirmed successful reconstruction of the whole ascending aorta and no progression of the other aortic lesion at 1 year after operation
Hebson et al./Short communication/ (8) /2014	6-months, F	Severe AA + DA	c. 376G>A, p. Glu126Lys HZ missense mutation	No	Stable thoracic aortic grafts at 2$\frac{1}{2}$years of age
Sawyer et al./Clinical report/ (11) /2013	Study on 4 related individuals	Surviving patient had dilation of AR	c. 376G>A, p. Glu126Lys (all 4), HZ, missense mutation non-synonymous	No	3 died, one long-term survival (boy 8-years old, when report published, underwent surgery at 22 months of age)
Erikson et al./Article/(12) /2012	31-week gestation, M infants (twins)	Postmortem gross findings identified marked vessel tortuosity	c. 85delG, HZ deletion mutation	No	Died
Kappanayil et al./Research article/(13)/2012	22 infants	All 22: dilation of the AA + AAr (Refer Table 2 in paper for entire spectrum of cardiac defects)	In 21/22 infants: c. 608A>C, p. Asp203Ala, HZ missense mutation. In 1/22 p. Arg227Cys compound HtZ substitution mutation	No	5/22 infants survive (when report published in 2012); Early mortality-−81%; none of the 22 infants underwent surgery
Renard et al./Research article/(14)/2010	20 years, F 7 years, M c) 18 months, F	AA; dilated AA; severe AA dilatation + severe tortuosity of the entire aorta	a) c. 376G>A, p. Glu126Lys, HZ missense mutation; b) c. 1189G>A, p. Ala397Thr, HZ missense mutation; c) c. 377A>T, 577delC, p. Glu126Val, c.577delC, compound HtZ missense +frameshift mutation	Yes	a) Stable, underwent surgery at 2.5, 7, and 8 months of age b) Asymptomatic—but no surgery, AA has increased to 54 mm c) Died
Hoyer et al./Short report/(15)/2009	Neonate F	Autopsy: thickened myocardium, bradycardia	c.800G>A, p. Cys267Thr, HZ missense mutation	Yes	Died at birth. Histology upon autopsy revealed increased vascular tortuosity
Dasouki et al./Research article/(16)/2007	Neonate F	Autopsy: dilation of AA	c. 835C > T (p.R279C)/c.1070_1073dupCCGC), compound HtZ missense + 4 base duplication	Yes	Died
Hucthagowder et al./Case report/ (17) /2006	2 years, F	AR	c. 169G>A, p, Glu57Lys, HZ missense mutation	No	Not specified

*AR, Aortic Root; AA, Ascending Aorta; AAr, Aortic Arch; DA, Descending Aorta; HZ, HomoZygous; HtZ, HeteroZygous; M, male; F, female; ARCL, Autosomal Recessive Cutis Laxa. ^#^Articles published in English language only*.

The role of fibulin-4 in elastic fiber formation is evolutionarily conserved in mammals. Additional observations in previous study, such as vascular tortuosity and bone fragility, indicate that fibulin-4 may have additional, pleiotropic functions in vascular patterning and collagen biosynthesis (17). As fibulin-4 deficiency has also been linked to abnormal smooth muscle cell differentiation and dysregulation of transforming growth factor beta signaling in the aorta, the specific vascular finding of “sausage link” appearance of the aneurysmal aorta and stenotic portion is suggested to be nearly universal in cases of this mutation (7, 13).

The differential diagnosis for several connective tissue disorders associated with arterial tortuosity and aneurysm formation show considerable clinical, histological, and pathophysiological overlap (11), and therefore, make the differential rather broad and confounding. Different genomic mapping/sequencing approaches have been suggested as the first line approach while investigating these genetically heterogenous disorders (6–8, 11). With only eight total *EFEMP2* mutations previously described (6), these can be associated with symptomatic aneurysms in early childhood and should be considered by the physician when evaluating an infant or young child with severe aortic dilation. Previous reports have described a high mortality rate associated with *EFEMP2* mutations until early childhood, likely as a result of aortic coarctation or the presence of an aneurysm and compression of airway or pulmonary artery (8, 11, 13). Homozygosity of mutant *EFEMP2* alleles in patients has been strongly linked to early presentation and high lethality (13). Despite these factors, the overall asymptomatic status at the time of admission and the longevity of our patient sets her clinically apart. It has been suggested that there may be a role for hitherto unknown factors apart from the physical deformation of arteries and coarctation, in determining mortality and morbidity (13).

Skin manifestations are more prominent in autosomal dominant, AR type 2 and AR type 1A cutis laxa types, whereas cardiac manifestations are more prominent in ARCL 1B (10), where redundant skin folds is less commonly observed (13). Although some patients with cutis laxa may have striking phenotypic features aiding in diagnosis (13, 15, 17), our patient did not present with any apparent skin abnormalities, as also shown in previous reports (6, 7, 11, 14, 17). Exon 5 mutations in *EFEMP2*, has been associated with minimal cutis laxa phenotypic features on presentation (6, 11, 17), but our patient exhibited the same phenotype with an exon 7 mutation. Whether specific exon aberrations correlate to cutis laxa phenotype severity, is subject to further investigations.

A previous detailed study involving 22 patients with an identical novel mutation in *EFEMP2*, has provided one of the largest clinical evidence for a vital role of fibulin-4 in human elastogenesis (13). The homozygous single base substitution (c.608A > C), identified in 21/22 probands was also identified in our patient. This single base substitution results in an Asp203Ala substitution that affects the highly conserved DVNE consensus sequence of the fourth calcium-binding epidermal growth factor domain. This consensus sequence is critical for calcium ion binding and is therefore, essential for the structural and binding properties of the fibulin-4 protein.

Our patient presented with AI. Progressive dilation of the ascending aneurysm may result in AI due to dilation of the aortic annulus. While her LV ejection fraction was normal, occurrence of AI causes a significant volume overload on the LV, and can result in progressive LV dilation and failure. Important factors to be considered as an indication for surgery include progressive AI, an aortic size over 5 cm, aneurysm growth rate exceeding 1 cm per year, and a genetic predisposition to early aortic dissection (3, 18). There is sparse information about the risk of aortic rupture or dissection in children or young adults, with no clear cut-off point regarding the diameter of aneurysm. As reported previously (19, 20), an index of >160% (ratio of the aneuric diameter to the normal diameter of the ascending aorta) was calculated for our patient that warranted a surgical intervention.

Because of the early lethal nature of the disease (11–13, 15, 16), an early diagnosis and intervention are required. Our patient only demonstrated the presence of an aneurysm, and as suggested previously, which may be associated with cerebral embolization (7). Close observation of the mediastinal structure is therefore important to determine the optimal timing of surgical repair (7). Recognizing the pattern of dilation permitted timely surgical intervention in our patient, with resection of all dilated portions of the aorta and therefore, minimized the chances of subsequent aortic dissection. Reports of surgical intervention in ARCL 1B patients are limited in part due to the death occurring prior to diagnosis (11). However, successful post-operative outcomes and long-term survival has been observed for all the 9 patients who underwent surgery (out of a total of 42 known cases of TAA due to ARCL 1B, Table 1). Although early surgical intervention may be lifesaving, close follow-up is required as progressive dilation or vascular stenoses may occur in the remaining portions of the native aorta (6). Furthermore, because there are few reports about the long-term outcomes of ARCL 1B after primary repair, life-long meticulous follow-up is mandatory.

Conduit replacement and the valve-sparing (David V) operation are the current favorable surgical treatment procedures. The David V procedure has shown to provide excellent long-term valve function and low rates of valve-related complications in the elective treatment of aortic root aneurysms (21), and has been identified as an ideal procedure for young patients with aortic root pathology (22). Homograft replacement is shown to have disappointing durability (20). Our patient was indicated for a valve-sparing operation because the nature of aortic valves was thought to be suitable for the aortic valve reimplantation. The aortic valves did not exhibit any relevant structural defect or severe cusp prolapse. While the Ross operation might be an alternative for patients with combined ascending aneurysms and severe aortic valve dysfunction, it is often reconsidered because of delayed problems associated with the autograft and the durability of the conduit in the pulmonary position (20). Bicuspidization technique can be used as a reconstructive approach for the regurgitant morphological tricuspid aortic valve with cusp retraction to avoid complications of anticoagulation (3). Likewise, aortic root replacement with aortic valve-sparing is attractive for children who have a normally functioning aortic valve because it may avoid anticoagulation (23).

Good perioperative and postoperative results can be achieved by early diagnosis and an assessment of the indications depending on the individual etiology. Pre- and perioperative care involves an intricate interplay of several key factors (24). Patients typically require close monitoring and observation in an intensive care unit, given the high risk of sudden hemodynamic instability. After surgery, the control and optimization of fluid balance essential, as well as a good pain control. Individual and valve-preserving treatment strategies have a favorable influence on the operative outcome, as observed in our case where the patient was discharged in healthy medical conditions after the cardio-intervention.

## Conclusions

Our report shows an uncommon occurrence of TAA (involving multiple aortic segments) with severe AI that was successfully repaired using the valve-sparing (David V) operation. The severe aortopathy was linked to a homozygous EFEMP2 mutation. Close preoperative and postoperative observation is warranted, given the structural frailty of the aortic wall in patients with this mutation along with mandatory life-long meticulous follow-up.

## Data Availability Statement

The original contributions presented in the study are included in the article/supplementary material, further inquiries can be directed to the corresponding author/s.

## Ethics Statement

Ethical review and approval was not required for the study on human participants in accordance with the local legislation and institutional requirements. Written informed consent was obtained from the individual(s), and minor(s)' legal guardian/next of kin, for the publication of any potentially identifiable images or data included in this article.

## Author Contributions

AV and PT collected the data. AV followed up on the patient till discharge. SN analyzed the literature and wrote the manuscript. SC provided radiological consultation. LC and JP helped in review of literature. TM, TF, and VG reviewed and edited the manuscript. All authors read and agreed to the final version of the manuscript.

## Conflict of Interest

The authors declare that the research was conducted in the absence of any commercial or financial relationships that could be construed as a potential conflict of interest.

## Publisher's Note

All claims expressed in this article are solely those of the authors and do not necessarily represent those of their affiliated organizations, or those of the publisher, the editors and the reviewers. Any product that may be evaluated in this article, or claim that may be made by its manufacturer, is not guaranteed or endorsed by the publisher.
